# Investigating the Long-term Effects of Misinformation, Disinformation, and Malinformation in the Health System

**DOI:** 10.34172/aim.34286

**Published:** 2025-08-01

**Authors:** Sedighe Sadat Tabatabaei Far, Milad Ahmadi Marzaleh

**Affiliations:** ^1^Student Research Committee, School of Health Management and Information Sciences, Shiraz University of Medical Sciences, Shiraz, Iran; ^2^School of Health Management and Information Sciences, Shiraz University of Medical Sciences, Shiraz, Iran

## Dear Editor,

 “Misinformation” is the dissemination of false information without the intention to mislead. Those who share this false information may believe that the information is true, useful, or interesting, and have no malicious intent toward the people they share it with.^1^

 Health “misinformation” can be divided into three different types based on its accuracy, each of which poses risks to individuals and society. The first type of misinformation involves the dissemination of *completely incorrect health information* that can lead to harmful health decisions. The second type of misinformation refers to the *spread of health pseudoscience*, where some elements of truth are presented in a misleading way, leading to incorrect conclusions. Finally, the third type of misinformation involves *conditional advice* of uncertain validity that, despite good intentions, can lead to negative health outcomes if used incorrectly. All three types can significantly harm public health and create confusion in health decision-making.^2^ For example, skepticism about the COVID-19 vaccine is prevalent in many communities, especially among groups with specific socioeconomic and racial backgrounds. Given the rapid development of this vaccine, many people have become skeptical about its effectiveness and safety. Stroud’s study showed that people who are able to identify inaccurate health articles are less likely to spread misinformation, have less skepticism about vaccines, and are more likely to get vaccinated.^3^

 “Disinformation” is designed or disseminated with full knowledge that it is false (the information has been manipulated) and is part of an intent to deceive and cause harm. The motives can be economic, ideological, religious, political or in support of a social agenda, etc. This type of information can also cause harm, including threats to decision-making processes as well as health, the environment or security. “Disinformation” in public health is a specific type of information risk that, unlike “misinformation”, is created with malicious intent to create discord and distrust in targets such as government institutions, scientific experts, public health institutions, the private sector and law enforcement agencies, etc.^1^

 The potential consequences of “disinformation” can be understood through examples from the COVID-19 pandemic. The COVID-19 pandemic had two key elements that created the perfect storm for the proliferation and spread of “disinformation.” First, it rapidly created fear, uncertainty, and doubt globally. Second, it occurred at a time when information (including misinformation and disinformation) was easily accessible, created, and shared via the internet, mobile communications, media, and social networking platforms. As the pandemic spread, many posts appeared on social media and were shared via instant messaging communications that increased uncertainty about treatment, the safety and effectiveness of vaccines, the application of social distancing, and so on. This situation led to social protests, unrest, delays in vaccine adoption, and in some cases, higher mortality rates.^1^

 In the age of social media and rapid information sharing, “misinformation” can spread rapidly during disasters. Authorities must proactively counter false narratives and “disinformation” campaigns that can undermine public safety or cause confusion.^4^

 Health “malinformation” has significant negative impacts on public trust in the health system. This type of information, which refers to valid medical information but in an incorrect context, can lead to false and misleading perceptions in society. In fact, health “malinformation” is deliberately disseminated with the intention of harming and can harm individuals and organizations. This information is presented without regard to the original context or in an incorrect context to lead people to incorrect conclusions. The dissemination of “malinformation” can lead to public distrust in health systems, especially at times of crisis such as pandemics. This can lead to reduced public cooperation in adhering to health protocols, and consequently, have negative impacts on public health. Furthermore, “malinformation” is difficult to detect due to the use of seemingly valid and misleading facts, which can lead to distrust in medical science and research.^5^ In fact, “malinformation” is based on fact but is shared with the intent to cause harm. This type of information typically involves the transmission of content that was intended to remain private but is intentionally made public to harm individuals, organizations, or countries.^6^

 An example of this type of information is the Los Angeles City Fire, which was not only met with misinformation, but also with disinformation and malformation aimed at creating panic, instability, or increasing damage in crises.^4^

 To provide contextual clarity, documented examples of misinformation, disinformation, and malinformation in the Iranian health system are presented below:

 Misinformation: According to a cross-sectional assessment by Hassanian-Moghaddam et al, by May 2, 2020, a total of 5876 individuals had been hospitalized and 534 had died due to methanol poisoning. Furthermore, the Iranian Legal Medicine Organization reported a total of 800 methanol-related deaths during the same period. These numbers represent one of the largest methanol poisoning outbreaks globally and underscore the life-threatening consequences of unverified health information.^7^

 Disinformation: According to a study by Taefehshokr et al, disinformation efforts significantly contributed to vaccine hesitancy in Iran and delayed the national vaccination campaign, leaving millions vulnerable to the virus.^8^

 Malinformation: A study by Mobasher et alhighlighted that breaches of patient privacy occurred in Iranian healthcare settings during the pandemic, underscoring the risks associated with the exposure of sensitive health information. The study shows that, in some cases, patients’ confidential information has been disclosed without authorization, which could be an example of malinformation. These disclosures may have been made with the intent to harm the reputation of specific healthcare institutions or professionals.^9^

 These examples underline the multifaceted nature of health-related information threats and the urgent need for effective monitoring and response mechanisms tailored to the Iranian context

 Prevention strategies for all three types of information are shown in Table 1.

**Table 1:** Prevention Strategies for All Three Types of Information

**Strategies to prevent "malinformation"**	**Strategies to prevent "disinformation"**	**Strategies to prevent "misinformation"**
1) Increasing information transparency: Providing accurate information 2) Encouraging dialogue: Having experts answer people’s questions 3) Using influencers: Working with celebrities to correct accurate information 4) Active monitoring: Tracking and identifying malinformation in the media 5) Building trust: Strengthening communication between the health system and the public 6) Collaborating with the media: Disseminating accurate information through credible media	1) Identifying Motivations: Countering the financial and political motives behind the dissemination of disinformation. 2) Public Education: Strengthening media literacy and critical thinking skills. 3) Technological tools: Developing algorithms to identify and remove disinformation 4) International cooperation: Building global coalitions to counter disinformation 5) Supporting independent media: Encouraging independent media to provide accurate and unbiased information.	1) Education and awareness raising: Educating people to identify and verify the accuracy of information. 2) Using credible sources: Verifying sources and ensuring their credibility. 3) Accurate information: Disseminating accurate and scientific information from credible sources. 4) Social platforms: Identifying and reporting misinformation on social media. 5) Personal responsibility: Refraining from disseminating unspecified information. 6) Laws and regulations: Enforcing laws to reduce the spread of misinformation.

## Conclusion

 The spread of misinformation in the health sector, particularly with the spread of social media, is divided into “misinformation”, “disinformation”, and “malinformation”, which can lead to incorrect decisions and reduce public trust. During the COVID-19 pandemic, this information caused social unrest and delays in vaccine adoption by creating fear and doubt. Health “disinformation” also poses serious risks to public health and trust in medical science, with the intention of causing harm and presenting facts in a false context.
